# Senicapoc in Patients with Idiopathic Pulmonary Fibrosis or Other Progressive Fibrotic Interstitial Lung Diseases: Protocol for a Randomised, Double-Blind, Placebo-Controlled, Multicentre Phase II Trial

**DOI:** 10.3390/diagnostics16111649

**Published:** 2026-05-27

**Authors:** Line Kølner-Augustson, Alan Altraja, Elisabeth Bendstrup, Peter Bradding, Nanna Makholm, Andrew M. Wilson, Ulf Simonsen, Ole Hilberg

**Affiliations:** 1 Department of Medicine, Lillebaelt Hospital, 7100 Vejle, Denmark; ole.hilberg@rsyd.dk; 2 Department of Regional Health Research, University of Southern Denmark, 5230 Odense M, Denmark; 3 Department of Pulmonology, Lung Clinic, Tartu University Hospital, 50411 Tartu, Estonia; 4 Department of Respiratory Diseases and Allergy, Aarhus University Hospital, 5200 Aarhus N, Denmark; 5 Department of Clinical Medicine, Aarhus University, 8000 Aarhus C, Denmark; 6 Department of Respiratory Sciences, University of Leicester, Leicester Respiratory NIHR BRC, Glenfield Hospital, Leicester LE1 7RH, UK; 7 Norwich Medical School, University of East Anglia, Norwich NR4 7TJ, UK; 8 Department of Biomedicine, Aarhus University, 5000 Aarhus C, Denmark

**Keywords:** idiopathic pulmonary fibrosis, progressive fibrotic interstitial lung disease, senicapoc, potassium channels, calcium-activated, antifibrotic agents, vital capacity, clinical trial protocol, drug repositioning, randomised controlled trial, translational medical research

## Abstract

**Background/Objectives**: Idiopathic pulmonary fibrosis (IPF) and other progressive fibrotic interstitial lung diseases (F-ILD) are characterised by progressive loss of lung function, worsening symptoms, and poor prognosis. Current antifibrotic therapies slow disease progression but do not arrest or reverse fibrosis and are frequently associated with adverse effects. Senicapoc, a selective KCa3.1 channel inhibitor, has shown antifibrotic effects in preclinical models, human lung myofibroblasts, and ex vivo human lung tissue. This study aims to determine whether senicapoc reduces the rate of decline in forced vital capacity (FVC) over 26 weeks, compared with placebo, in patients with IPF or other progressive F-ILD, while also characterising safety and tolerability. **Methods**: This is an investigator-initiated, prospective, randomised, double-blind, placebo-controlled, multicentre phase II trial. Adults with IPF or other F-ILD with documented progression despite optimised antifibrotic management according to standard care and individual tolerability will be randomised 1:1 to receive senicapoc 30 mg once daily or a matching placebo for 26 weeks in addition to standard care. The primary outcome is the rate of decline in FVC over 26 weeks. Secondary outcomes include changes in diffusion capacity, 6 min walk distance, dyspnoea, health-related quality of life, adverse events, and senicapoc plasma concentrations, with mortality and exacerbations assessed as exploratory outcomes. The planned sample size is 140 participants. The primary analysis will be conducted in the intention-to-treat population using a linear mixed-effects model for repeated measurements. **Results**: No results are available, as this article describes the study protocol. **Conclusions**: This study will provide proof of concept for the efficacy, safety, and tolerability of senicapoc in progressive fibrotic interstitial lung disease. If successful, it will support further clinical development of KCa3.1 inhibition as a novel antifibrotic strategy.

## 1. Introduction

Idiopathic pulmonary fibrosis (IPF) is a chronic, progressive fibrotic interstitial lung disease that occurs predominantly in former smokers and is typically diagnosed in patients aged 70 years or older [1]. The disease is characterised by worsening dyspnoea, progressive loss of lung function and poor survival and is viewed as the prototypical progressive fibrotic interstitial lung disease [1,2]. However, a proportion of patients with other fibrotic interstitial lung diseases (F-ILDs) also develop progressive disease despite appropriate management, including the avoidance of relevant triggers and immunosuppressive treatment where indicated [1,3]. These patients are now recognised as having progressive pulmonary fibrosis, a clinical phenotype defined by ongoing fibrotic progression irrespective of the underlying interstitial lung disease subtype [1].

Pulmonary fibrosis arises from repetitive epithelial injury, followed by aberrant wound healing rather than normal tissue generation. Dysfunctional alveolar epithelial cells release profibrotic mediators, including transforming growth factor beta 1 (TGF-β1), which promote fibroblast recruitment, activation and proliferation, culminating in differentiation into myofibroblasts. These cells deposit excessive extracellular matrix, leading to architectural distortion, progressive loss of lung compliance, impaired gas exchange, and ultimately respiratory failure [2,4].

Over the past decade, substantial research has sought to address the unmet need for effective treatment of these conditions. Three antifibrotic agents, pirfenidone, nintedanib and nerandomilast, are approved in the European Union, the United States, and elsewhere and constitute standard care for many patients [1,5,6,7,8]. Clinical studies have shown that these agents slow disease progression in other progressive F-ILD, broadly consistent with their effects in IPF [5,6,9,10,11]. However, these therapies do not arrest or reverse disease progression, likely reflecting the complexity of profibrotic pathways, in which antifibrotics modulate aspects of profibrotic signalling and fibroblast activity but not the already established extracellular matrix remodelling and architectural distortion. Current therapies also have considerable limitations in tolerability. Pirfenidone is associated with gastrointestinal symptoms, fatigue, rash and photosensitivity [5], whereas nintedanib is associated with diarrhoea, nausea, weight loss and liver enzyme abnormalities [6]. In clinical practice, these adverse effects frequently lead to dose reduction, temporary interruption or treatment discontinuation. Therefore, there remains a need for novel therapies that target disease-relevant pathways, provide greater antifibrotic efficacy or have a more favourable tolerability profile.

Senicapoc is an orally administered small molecule, also known as ICA-17043 (chemical name: 2,2-bis(4-fluorophenyl)-2-phenylacetamide), and a selective and highly potent inhibitor of the KCa3.1 channel. The KCa3.1 channel is an intermediate-conductance, calcium-activated potassium channel, also known as IK1, SK4, IKCa1, or Gardos channel. It is encoded by the KCNN4 gene and plays a critical role in regulating membrane potential and calcium homeostasis in immune cells, fibroblasts, endothelial cells, and cancer cells [12,13,14]. Senicapoc inhibits KCa3.1-mediated K^+^ efflux with high potency, with an IC50 of 6–11 nM [12]. Importantly, senicapoc has minimal effects on other K^+^ and Na^+^ channels, including those in cardiac tissue, even at concentrations 900-fold higher than those required to inhibit the KCa3.1 channel [15]. Senicapoc was originally developed for sickle cell anaemia, in which erythrocyte dehydration during sickling is primarily driven by K^+^ efflux through KCa3.1 channels [16]. Although the phase III trial was discontinued because the primary clinical endpoint, reduction in vaso-occlusive crises, was not met, treatment was associated with clear biological effects, including improvements in haemoglobin and markers of haemolysis [17]. These studies provided important human safety data and showed oral bioavailability suitable for once-daily dosing and a mean half-life of approximately 12.8 days [14]. This supports further clinical development in other indications.

The KCa3.1 channel is involved in Ca^2+^-dependent signalling and membrane potential regulation and contributes to several fibroblast functions relevant to fibrogenesis, including activation, migration, and proliferation. As these processes are central to tissue fibrogenesis, the KCa3.1 channel may represent a relevant therapeutic target in fibrotic diseases, including pulmonary fibrosis [18,19,20,21,22,23,24,25]. In a bleomycin-induced sheep model of pulmonary fibrosis, senicapoc treatment reversed reductions in lung compliance and reduced extracellular matrix accumulation, collagen deposition, and α-smooth muscle actin (α-SMA) expression in myofibroblasts [23]. In the same model, senicapoc attenuated increases in blood vessel density, vascular endothelial growth factor expression, and endothelial cell proliferation, with effects that appeared greater than those observed with pirfenidone [24]. In addition, KCa3.1 channel blockade alleviated endoplasmic reticulum stress and apoptosis in type II alveolar epithelial cells and macrophages, which may have contributed to the observed antifibrotic effect [25]. Beyond the lung, renal fibrosis has likewise been shown to be attenuated by targeted disruption of KCa3.1 channels [26], and TRAM-34, a KCa3.1 inhibitor closely related to senicapoc, suppresses fibroblast proliferation in paraquat-induced pulmonary fibrosis [27].

Further support for the antifibrotic potential of senicapoc comes from studies in human lung myofibroblasts and ex vivo human lung slices. Compared with fibroblasts from non-fibrotic lungs, IPF-derived human lung myofibroblasts show higher KCa3.1 expression and greater KCa3.1-sensitive current [18]. In these cells, senicapoc attenuates TGF-β1- and basic fibroblast growth factor-dependent profibrotic activity and promotes dedifferentiation towards a quiescent fibroblast phenotype [20]. This is particularly relevant because TGF-β1 is a key mediator of fibrogenesis in IPF [22]. In cultured human lung slices derived from both non-fibrotic and IPF lungs, TGF-β1 induced broad profibrotic transcriptional and protein-level changes, including the upregulation of 41 fibrosis-associated genes, increased extracellular matrix and fibroblast-specific protein expression, and enhanced collagen secretion. Many of these TGF-β1-induced changes were attenuated by senicapoc but not by dexamethasone [21,28]. In another human lung slice model, pirfenidone and nintedanib reduced TGF-β1-induced fibroblast proliferation, whereas only senicapoc prevented the accompanying increase in tissue α-SMA expression [29]. Taken together, findings from studies in animals, human lung myofibroblasts, and ex vivo human lung tissue all support an antifibrotic effect of senicapoc and justify further investigation in fibrotic lung disease.

In addition to sickle cell anaemia, senicapoc has been investigated in patients with COVID-19-related respiratory insufficiency [30] and in asthma [13]. Across previous clinical studies, no major safety signals attributable to senicapoc have been identified. Treatment-emergent adverse events reported more frequently with senicapoc than placebo have included nausea (16% vs. 10%), urinary tract infection (14% vs. 8%) and sickle cell crisis-related events [15,17,31]. Of particular relevance to repurposing senicapoc for chronic lung disease, no between-group differences in corrected QT interval have been reported, although one sickle cell disease trial found an isolated increase in gamma-glutamyl transferase [17].

Taken together, the available mechanistic, preclinical, translational, and clinical safety data provide a strong rationale for evaluating senicapoc as a novel antifibrotic treatment in IPF and other progressive F-ILD. The present study aims to determine whether senicapoc reduces the rate of decline in forced vital capacity (FVC) over 26 weeks compared with placebo in patients with IPF or other progressive F-ILD, while also characterising its safety and tolerability.

## 2. Materials and Methods

### 2.1. Study Design and Setting

This investigator-initiated, prospective, randomised, double-blind, placebo-controlled, multicentre phase II trial is designed to evaluate the efficacy and safety of senicapoc, in addition to standard care, in adults with IPF or other progressive F-ILD. The primary objective is to determine whether senicapoc reduces the rate of decline in FVC (mL) over 26 weeks compared with placebo. Patients and the public were not involved in the design, conduct, reporting, or dissemination planning of this trial.

The trial is coordinated by the Department of Medicine, Lillebaelt Hospital, Vejle, Denmark, in collaboration with academic and tertiary ILD centres in Denmark, Estonia and the United Kingdom. Recruitment and study procedures will be conducted in specialist outpatient ILD clinics with experience in the diagnosis and longitudinal management of IPF and progressive fibrotic ILD. Trial conduct will be overseen by the sponsor and the national Good Clinical Practice units, with independent statistical analysis performed after database lock.

### 2.2. Patient Population

Eligible patients are adults aged 18 years or older with IPF or other progressive F-ILD and documented progression despite optimised antifibrotic management.

### 2.3. Inclusion Criteria

Participants must meet all of the following criteria:

- Age of 18 years or older.
- Diagnosis of IPF or other progressive F-ILD according to the applicable ATS/ERS/JRS/ALAT guidelines at the time of diagnosis [1].
- Receipt of optimised antifibrotic treatment according to standard care and individual tolerability. This may include nintedanib, pirfenidone, nerandomilast, reduced-dose treatment, or no antifibrotic therapy if treatment is contraindicated, not tolerated due to adverse effects, or declined by the participant.
- Documented disease progression despite optimised antifibrotic treatment, defined as an annual absolute decline in FVC of at least 5% [1], based on at least three measurements obtained 6–24 months before enrolment.
- High-resolution computed tomography performed within 24 months before enrolment, demonstrating fibrotic changes exceeding the extent of emphysema.
- FVC greater than 45% predicted and FEV1/FVC greater than 0.7 or above the lower limit of normal.
- Ability to walk at least 150 m during the 6 min walk test and complete study questionnaires.
- Stable condition, except progression in F-ILD, and suitable for study participation based on medical history, physical examination, electrocardiography, and laboratory assessment.
- Ability and willingness to comply with study procedures, including contraceptive requirements where applicable.

### 2.4. Exclusion Criteria

Participants will be excluded for any of the following:

- Participation in another investigational study or exposure to another investigational medicinal product (IMP) for F-ILD within the previous 6 months.
- Known hypersensitivity to senicapoc or any of the excipients, or a history of a significant allergic reaction to any drug.
- Clinically significant immunosuppressive conditions (e.g., human immunodeficiency virus infection, congenital, acquired, medication-induced), sickle cell disease, or current alcohol or substance misuse.
- Malignancy within the previous 5 years, except for squamous cell carcinoma, uterine cervix carcinoma in situ, treated basal cell carcinoma of the skin without recurrence or prostate cancer managed through watchful waiting.
- Clinically significant electrocardiography abnormalities or known long QT syndrome.
- Previous lung volume reduction surgery or lung transplantation.
- Severe pulmonary hypertension or unstable major cardiovascular, pulmonary, or other systemic disease.
- Moderate to severe hepatic impairment, cholestatic disease or clinically relevant liver biochemistry abnormalities, or creatinine clearance below 30 mL/min.
- Current or planned use of any of the following therapies during the study: warfarin, imatinib, ambrisentan, azathioprine, cyclophosphamide, ciclosporin, bosentan, methotrexate, sildenafil (except for erectile dysfunction), and prednisone at a stable dose greater than 10 mg/day.
- Lower respiratory tract infection requiring treatment within 4 weeks of screening.

### 2.5. Recruitment and Consent

Participants will be recruited from outpatient clinics at tertiary care institutions in Denmark, Estonia and the United Kingdom. Potential participants will be identified during routine outpatient follow-up, and investigators will provide verbal and written information about the trial. A deliberation period of at least 24 h will be provided, unless the participant requests otherwise and local regulations permit. For patients who choose to participate, the enrolment visit will be scheduled within a few days.

### 2.6. Study Treatment

**Figure 1:**
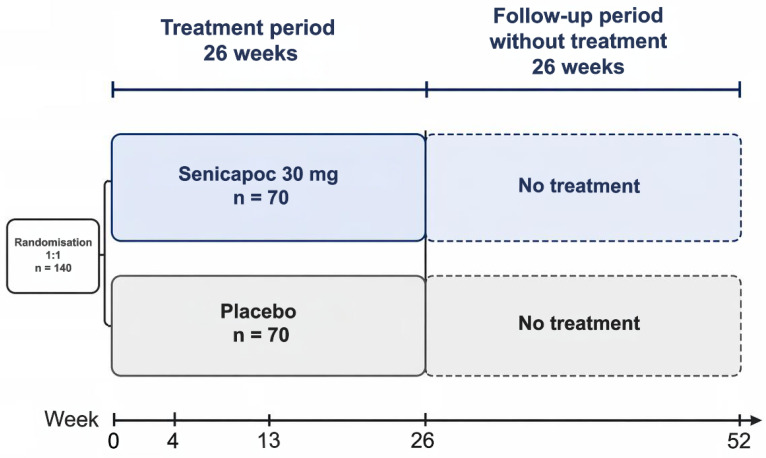
Trial design. Participants will be randomised 1:1 to receive senicapoc 30 mg once daily or matching placebo for 26 weeks, shown in blue and grey, respectively. Dashed boxes indicate the 26-week follow-up period without study treatment. The planned sample size is 140 participants, with 70 participants allocated to each treatment arm. Study visits are scheduled at baseline/randomisation and at weeks 4, 13, 26 and 52.

Participants will receive either senicapoc 30 mg once daily, administered as three 10 mg tablets, or a matching placebo for 26 weeks, in addition to standard care. Trial design is shown in Figure 1. Dose selection was informed by prior clinical data and ongoing studies and is expected to achieve a steady-state free plasma concentration of approximately 30 nM, which is considered sufficient for KCa3.1 channel inhibition [12,14].

Placebo tablets will match senicapoc tablets in size, colour, and excipient composition but will not contain the active ingredient. Dose modification will not be permitted.

Study medication will be dispensed at treatment initiation in five labelled packs. Each participant will receive sufficient tablets to complete 26 weeks of treatment. Participants will be instructed to return all unused tablets at the end-of-treatment visit. Tablet counts will be used to assess adherence, and any unused medication will be handled in accordance with regulatory requirements.

Manufacturing, quality control, release, stability testing and packaging is performed by STA pharmaceutical, Shanghai, China. Scantox, Ballerup, Denmark will conduct import, clinical packaging, labeling and shipment. Scantox is responsible for the release to study sites in accordance with national regulatory requirements, and is working with Myonex, Ltd, Leicester, UK, who are responsible for importation and distribution in the UK. 

### 2.7. Randomisation and Blinding

Participants will be randomised individually in a 1:1 ratio to receive either senicapoc plus standard care or placebo plus standard care. Randomisation will be performed in REDCap and stratified by study site and diagnosis (IPF or other progressive F-ILD). The allocation sequence will be generated electronically within the REDCap randomisation module and will be accessible only to the REDCap administrator. Investigators and the sponsor will not have access to the allocation sequence.

The trial will be double-blind. Participants, investigators, study personnel involved in participant care or assessment, outcome assessors, and data analysts will remain blinded to treatment allocation throughout the trial. Blinding will be maintained by using matching placebo tablets identical in appearance to the active treatment.

Following randomisation, the participant’s identification number and date of randomisation will be recorded. Once assigned, participant identification numbers and treatment allocations cannot be reassigned. Site personnel will maintain a confidential participant identification log in accordance with regulatory requirements.

The treatment allocation code will be stored in REDCap, and all access will be recorded in an audit trail. Emergency unblinding will be available when knowledge of the treatment is necessary for the clinical management or safety of an individual participant. In such cases, the principal investigator may request unblinding, and the reason for unblinding will be documented.

### 2.8. Study Procedures and Visits

The trial will include five study contacts, including enrolment. Before randomisation, written informed consent must be obtained and eligibility confirmed. Investigators and trained study personnel will collect study data and enter them into the REDCap database. An overview of study visits and procedures is provided in Table 1.

At baseline, the following procedures will be performed: spirometry and diffusing capacity (DLCO); 6 min walk distance; electrocardiography; physical examination; blood sampling; and patient-reported outcome measures. High-resolution computed tomography will be performed if no suitable scan has been obtained within the preceding 24 months. In addition, demographic information, comorbidities and baseline medication, including current antifibrotic treatment, will be obtained from the medical records. Assessments performed within 1 month may be used as a baseline where appropriate.

The 4-week visit is a safety visit and will include blood sampling, visual analogue scale (VAS) of dyspnoea, and assessment of adverse events and treatment tolerability.

The 13-week visit will include spirometry and diffusing capacity of the lung (DLCO), physical examination, patient-reported outcome measures, and assessment of adverse events and treatment tolerability.

The 26-week visit will include spirometry and DLCO, 6 min walk distance, electrocardiography, physical examination, blood sampling, patient-reported outcome measures, and assessment of adverse events and treatment tolerability.

The 52-week visit will be conducted during a routine outpatient follow-up clinic consultation. It will consist of safety blood sampling and a physician consultation to assess the washout period, including assessment of adverse events and treatment tolerability.

Blood samples will be collected for safety monitoring. Safety laboratory assessments will include C-reactive protein, white blood cell count with differential, haemoglobin, platelet count, sodium, potassium, calcium, serum creatinine, estimated glomerular filtration rate (eGFR), aspartate aminotransferase, alanine aminotransferase, total bilirubin, gamma-glutamyl transferase, and total serum bile acid concentration. Additional blood samples to measure senicapoc concentration will be collected at weeks 4 and 26. These samples will be centrifuged, and serum will be frozen until analysis after the final visit of the last patient. Any further use of stored biological material will require relevant regulatory approval and renewed participant consent. Remaining material will be destroyed after completion of the planned analyses.

To minimise participant burden, blood sampling will be restricted to predefined safety and pharmacokinetic assessments, coordinated with scheduled study visits and routine clinical blood tests, and performed by trained clinical personnel. Sample volumes will be kept to the minimum required for the analyses.

### 2.9. Outcomes

The primary endpoint is the rate of decline in FVC (mL) over 26 weeks.

Secondary outcomes include lung function and functional outcomes, namely changes in FVC (% predicted) and 6 min walk distance; symptom and health-related quality-of-life measures, including changes in the dyspnoea VAS, the EuroQOL 5-Dimensions Questionnaire [32], the St George’s Respiratory Questionnaire for Idiopathic Pulmonary Fibrosis [33], and King’s Brief Interstitial Lung Disease questionnaire [34]; safety outcomes, including the number and type of adverse events and plasma concentration of senicapoc; and exploratory clinical outcomes, including all-cause mortality and exacerbations. Secondary outcomes will be assessed over the 26-week treatment period, unless otherwise specified.

### 2.10. Safety Assessments

Adverse events and serious adverse events will be collected systematically from the first administration of study treatment until the end of follow-up or death, regardless of suspected relationship to the study treatment. At each contact, the investigator will assess adverse events through direct questioning and, where appropriate, clinical examination. Adverse events will be graded according to the Common Terminology Criteria for Adverse Events, version 5.0 [35]. Serious adverse events and suspected unexpected serious adverse reactions will be handled and reported in accordance with applicable regulatory requirements. Ongoing events at the end of follow-up will be followed until resolution, stabilisation, or outcome determination, as appropriate. The investigator will decide whether study treatment should be interrupted or discontinued in response to an adverse event.

### 2.11. Sample Size

The planned sample size is 140 participants, with 70 allocated to each treatment arm. This sample size was determined during trial planning and is specified in the approved study protocol. It was informed by FVC decline reported in prior studies and by treatment effects observed in antifibrotic trials [5,6,10,11], recognising that these assumptions may not fully reflect the present enriched population receiving contemporary standard care. The calculation assumes an FVC decline of 175 mL in the placebo group and 120 mL in the senicapoc group over 26 weeks, yielding a sample size of 62 participants per group (two-sided α = 0.05; power = 80%), and accounts for a 5% mortality rate during the 26 weeks [36].

### 2.12. Statistical Analysis

All efficacy analyses will be conducted primarily in the intention-to-treat population, defined as all randomised participants analysed according to their allocated treatment group. A per-protocol analysis will be performed as a sensitivity analysis and will include participants without major protocol deviations. Safety analyses will include all participants who receive at least one dose of study medication.

The primary endpoint, the rate of decline in FVC (mL) over 26 weeks, will be analysed using a linear mixed-effects model for repeated measurements. Fixed effects will include treatment group, study visit, treatment-by-visit interaction, and baseline FVC, with participant included as a random effect to account for within-participant correlation. The treatment effect will be reported as the adjusted between-group difference in FVC decline over 26 weeks, with 95% confidence intervals and two-sided *p*-values.

Exploratory analyses of the primary endpoint will be performed according to baseline antifibrotic treatment. These analyses will use two separate grouping strategies: one based on the specific antifibrotic therapy, and one based on treatment intensity. Where numbers permit, treatment-by-background therapy interaction terms will be explored. Exploratory subgroup analyses will also be performed according to diagnostic group, comparing participants with IPF and those with other progressive F-ILD. As randomisation is stratified by diagnosis, these analyses will examine the consistency of the estimated treatment effect across diagnostic strata.

Secondary continuous outcomes, including FVC % predicted, 6 min walk distance, dyspnoea VAS, and patient-reported outcome measures, will be analysed using mixed-effects models adjusted for baseline values. Binary outcomes will be analysed with logistic regression or other appropriate generalised linear models. Time-to-event outcomes, where relevant, will be analysed with Kaplan–Meier methods and Cox proportional hazards regression. Mortality outcomes will be summarised descriptively and interpreted as exploratory.

Adverse events will be summarised by treatment group, severity, background antifibrotic treatment and relationship to the study treatment. Laboratory safety variables will be summarised descriptively over time. All statistical tests will be two-sided, and *p*-values below 0.05 will be considered statistically significant for the primary endpoint. Secondary analyses will be considered supportive and exploratory, and no formal adjustment for multiple comparisons is planned.

Since the primary outcome is not mortality, no formal efficacy stopping criteria are defined. Given the relatively small sample size, no predefined futility stopping criteria are planned. No interim analyses will be performed.

The final statistical analyses will be conducted by an independent statistician using validated statistical software appropriate for the planned analyses. To ensure reproducibility without restricting the analysis to software that may be outdated by the time of database lock, the final statistical analysis plan will prespecify the software, version, and relevant packages or modules to be used. These details will be reported in the primary trial publication.

### 2.13. Missing Data and Intercurrent Events

Every effort will be made to minimise missing data, and outcome data will continue to be collected after treatment discontinuation wherever feasible. The extent, timing, and reasons for missing data will be summarised by treatment group. Sensitivity analyses may be performed if the extent of missing data is considered clinically relevant. For the primary endpoint, the likelihood-based mixed-effects model will include all available FVC measurements up to week 26. Missing FVC measurements for participants who remain alive will be handled within a likelihood-based mixed-model framework, assuming data is missing at random conditional on the observed data.

Deaths before week 26 will not be treated as ordinary missing data, as they preclude subsequent assessment of lung function. Deaths will be recorded and reported separately. Sensitivity analyses will be conducted to assess the robustness of the primary findings to informative truncation due to death. These may include a composite or worst-rank approach in which participants who die before week 26 are assigned the least favourable outcome and, where appropriate, additional analyses using multiple imputation for non-fatal missing data only.

### 2.14. Data Management and Sharing

Data will be collected by investigators and trained members of the research teams. Relevant data will be obtained from electronic medical records, laboratory assessments and patient-reported outcome measures. Each data entry will be linked to a unique study identification number and time-stamped. Source documents will be retained for data validation and quality assurance. Study data will be recorded and managed using REDCap, with role-based user access, audit trails, and data validation procedures. Study data will be stored securely within a protected analysis environment and retained in accordance with applicable regulatory requirements.

De-identified data will be made available 6 months after publication of the primary study results. Appropriate procedures will be applied before data sharing to minimise the risk of participant re-identification. Relevant trial documents, including the study protocol, data dictionary, and main statistical code, will be made available alongside the dataset. No predefined end date for data availability is planned. Data may be requested for scientifically sound research projects, subject to approval by an independent review committee. Data requests should be directed to the coordinating investigator. Requests will be evaluated by the trial steering committee based on methodological quality.

## 3. Discussion

This trial is designed to evaluate senicapoc as a potential antifibrotic treatment in patients with IPF and other progressive F-ILD. Both populations are included because, despite differences in underlying causes, these conditions share a pattern of progressive fibrosis, characterised by ongoing loss of lung function [1]. In addition, previous antifibrotic treatments have been shown to slow down disease progression in both IPF and other progressive F-ILD [5,6,9,10,11]. Since the approval of nintedanib for progressive fibrotic interstitial lung disease, this progressive phenotype has gained increasing clinical and therapeutic relevance. A treatment targeting a disease-relevant profibrotic pathway may be applicable across diagnostic subgroups, particularly when fibroblast activation, proliferation, and persistence are central to disease progression.

This trial evaluates senicapoc in addition to standard care, rather than replacing existing antifibrotic therapies. This is important because currently available antifibrotic agents do not stop or reverse disease progression, and many patients continue to worsen despite these treatments. Selecting patients whose condition is worsening despite optimised antifibrotic management yields a study population with active progressive disease. This allows evaluation of the antifibrotic effect of senicapoc without withholding standard care and may improve sensitivity for detecting a treatment effect. In this respect, the trial differs from previous IPF trials, which did not require documented disease progression before enrolment [5,6,8], and previous progressive F-ILD trials, which accepted progression documented within the preceding 24 months [7,9,10].

The trial uses the rate of decline in FVC over 26 weeks as the primary endpoint. This is appropriate because FVC decline is a well-established marker of disease progression in fibrotic interstitial lung disease and has been widely used as a primary endpoint in clinical trials [9,10,11]. In an early-phase setting, assessment of FVC decline over 26 weeks provides a clinically relevant and feasible means of identifying a potential treatment signal. This approach enables assessment of physiological disease progression over a timeframe suitable for a phase II study, without requiring the much larger sample sizes and extended monitoring periods needed to capture outcomes such as death, hospitalisation, or acute exacerbations.

Several limitations should be acknowledged. First, the trial is not powered to detect differences in less frequent but clinically important outcomes such as mortality, hospitalisation, or acute exacerbations. Second, including both IPF and other progressive F-ILD introduces clinical heterogeneity. Although this reflects current ILD practice and broadens the applicability of the findings to patients with progressive fibrotic lung disease, it may also increase variability in disease behaviour and treatment effect. The trial is not powered to assess treatment differences among specific ILD subtypes. Third, the blinded treatment and follow-up period are relatively brief. Although 26 weeks is appropriate for detecting early physiological signals, this timeframe may not fully capture the magnitude, durability, or longer-term clinical relevance of any treatment effect. Notably, this study cannot determine whether a reduction in FVC decline over 26 weeks leads to sustained benefits in symptoms, functional status, exacerbations, or survival. These questions will require evaluation in larger trials with longer follow-up.

Despite these limitations, the trial has several strengths. It evaluates a novel, biologically plausible therapeutic target, supported by convergent evidence from animal models and ex vivo human lung tissue. The randomised, double-blind, placebo-controlled design reduces bias in efficacy and safety assessment, and the multicentre recruitment across specialist ILD settings improves generalisability. In addition, restricting eligibility to patients with documented progression despite optimised standard care may increase sensitivity to detect a treatment signal. Findings that would support progression to a phase III trial include a reduction in FVC decline compared with placebo, the absence of major safety concerns, acceptable treatment tolerability, and supportive trends in secondary outcomes.

In summary, the trial addresses an important unmet need in fibrotic interstitial lung disease. Current treatments only slow disease progression, so new therapies with different mechanisms are needed. Senicapoc is promising based on its mechanism, preclinical results, and prior safety data.

## Figures and Tables

**Table 1:** Schedule of enrolment, randomisation, intervention, and assessments.

	Trial Period					
**Timepoint**	Screening (−t_i_ to 0)	Enrolment (0) *	Week 4 (±7 Days)	Week 13 (±7 Days)	Week 26 (±7 Days)	Week 52 (±30 Days)
Enrolment						
Eligibility assessment	X					
Informed consent	X					
Randomisation		X				
Intervention						
Senicapoc or placebo		Continuous daily treatment				
Assessments						
Demographics, comorbidities, and baseline medication		X				
Clinical examination		X		X	X	X
Spirometry		X		X	X	
Diffusion capacity		X		X	X	
6 min walk distance		X			X	
High-resolution computed tomography **		X				
Electrocardiography		X			X	
Safety assessment, blood sample		X	X		X	X
Biobank, blood sample			X		X	
EuroQOL 5-Dimensions Questionnaire		X		X	X	
St George’s Respiratory Questionnaire for Idiopathic Pulmonary Fibrosis		X		X	X	
King’s Brief Interstitial Lung Disease questionnaire		X		X	X	
Visual analogue scale of dyspnoea		X	X	X	X	
Adverse events/treatment tolerability assessment			X	X	X	X

X indicates that the procedure or assessment is performed at the corresponding timepoint. * Baseline data may be obtained within ±1 month of the enrolment visit, where applicable. ** Only performed if no scan has been obtained within the preceding 24 months.

## Data Availability

All relevant trial-related documents, including the protocol, investigators brochure and IMPD is available at EU Clinical Trials Regulation system (CTIS, number 2024-511131-97-00). Six months after the publication of the results, all de-identified individual patient data will be made available for data sharing. Procedures, including re-coding of key variables, will be put in place to allow for complete de-identification of the data.

## Abbreviations

The following abbreviations are used in this manuscript:

IPF	Idiopathic pulmonary fibrosis
F-ILD	Fibrotic interstitial lung diseases
FVC	Forced vital capacity
α-SMA	α-smooth muscle actin
TGF-β1	Transforming growth factor beta 1
FEV1/FVC	Ratio between Forced Expiratory Volume in one second and Forced Vital Capacity
